# The role of cerebellar impairment in emotion processing: a case study

**DOI:** 10.1186/s40673-018-0090-1

**Published:** 2018-10-12

**Authors:** Alexandra K Gold, Rosemary Toomey

**Affiliations:** Department of Psychological and Brain Sciences, Boston University, 900 Commonwealth Avenue, 2nd Floor, Boston, MA USA

**Keywords:** Cerebellum, Emotion, Obsessive-compulsive personality disorder

## Abstract

**Background:**

Though the cerebellum’s role in visuospatial and fine motor functioning has been well-established over the last several years, the role of the cerebellum in emotion has more recently been a focus of scientific inquiry. Cerebellar impairment has been associated with deficits in emotional processing and is linked to a wide range of clinical behaviors including social withdrawal, blunted emotional expression, and impulsivity. In addition, cerebellar impairments have been associated with the onset of psychiatric disorders including major depressive disorder and, more recently, obsessive-compulsive disorder.

**Case presentation:**

We describe a 32-year-old patient who presented to our clinic for a neuropsychological evaluation with a childhood history of a cerebellar brain tumor and detail-oriented, perfectionistic tendencies. Neuropsychological assessment data revealed impairments in visuospatial processing and in fine motor skills, likely stemming from the cerebellar tumor. Clinical assessment led to a diagnosis of obsessive-compulsive personality disorder and also suggested impairments in socio-emotional processing.

**Conclusions:**

Our findings lend support to recent data which has suggested the impact of cerebellar impairment on emotional processing and related domains. Unlike many previous studies, however, our report focuses on an individual who, despite having marked impairments in certain domains, demonstrates a high level of functioning. We believe that this report holds important clinical relevance for proper diagnosis of cerebellar-related impairment and for the necessity of early intervention.

## Background

Given the cerebellum’s implicated role in motor control and spatial navigation, a vast literature has linked cerebellar impairments to reduced visuospatial capacities and loss of fine motor functioning [1–5]. Over the past several years, many researchers have begun to explore the cerebellum’s role across varied domains of emotion function including emotional recognition, emotional learning, and emotional processing [6, 7]. Findings on the contributions of the cerebellum to an array of emotion domains have been observed in healthy and clinical populations. One recent study in healthy individuals found that applying transcranial magnetic stimulation over the left cerebellum (e.g., portion of cerebellum thought to be involved in emotional processing) affected participants’ abilities to accurately label emotional expressions and to specify the gender of emotional faces whereas neutral expressions and faces were unaffected [8]. Clinically, uncharacteristic emotional features resulting from cerebellar damage have been noted such as disinhibited behavioral responses and impulsivity in a patient with a right cerebellar infarction [9], stubbornness, thought rigidity, and inappropriate behavior (e.g., indecent humor) in patients with right-sided cerebellar lesions [7], and blunted emotional affect, social withdrawal, and low motivation in patients with left-sided cerebellar lesions [7]. In an extension of these clinical data, one study inferred that patients with cerebellar lesions may have a poor comprehension of their own emotional state because they did not report mood symptoms that they were observed to exhibit [10]. Some data suggest that cerebellar damage occurring earlier in life may result in more readily apparent emotion deficits by impacting normative growth processes such as social development and communication [11]. Notably, emotional and behavioral deficits resulting from cerebellar impairments may stem from excessive or restricted responses to one’s internal or external surroundings [12].

The emotional changes associated with cerebellar impairment have also been linked to the onset of several psychiatric conditions including depressive disorders, obsessive-compulsive disorder (OCD), and borderline personality disorder [12–15]. Whereas the association between cerebellar damage and major depression is well-documented [13, 16–19], only recently have studies begun to posit OCD pathology as stemming from impairments to the cerebellum. Within the past four years, case studies of patients with late-onset OCD hypothesize that left cerebellar lesions and cerebellar volumetric deficits may be implicating factors in OCD pathology [12, 20]. In this case presentation, we describe a patient with a childhood cerebellar tumor who presented to our clinic for neuropsychological evaluation. Her symptom presentation was reflective of deficits in socio-emotional processing and obsessive-compulsive tendencies.

## Case presentation

### Clinical history

Patient was a 32-year-old Caucasian female graduate student who self-referred to the Boston University Center for Anxiety and Related Disorders for a neuropsychological evaluation in order to renew the academic accommodations that she reported having for the entirety of her education. These accommodations included having assistance with academic work requiring the use of fine motor skills, having access to someone who could take notes for her (e.g., to limit the amount of writing she needed to complete), being in a distraction-free environment, and having extra time for assignments. Prior to beginning assessment procedures, the patient reviewed and signed the informed consent form, which was approved by the Boston University Institutional Review Board. Patient reported that her need for academic accommodations stemmed from a childhood cerebellar tumor which necessitated several associated procedures from the ages of 4–12. After the first appearance of the cerebellar tumor when the patient was 4 years of age, the patient was required to undergo an 8-h surgery to remove the tumor. Post-surgery hydrocephalus necessitated the placement of a ventral peritoneal shunt. Following this initial surgical procedure, the patient had right-sided paralysis, was wheelchair-bound for several months, and diagnosed with right hemiparesis, requiring her to become left-handed (e.g., in line with data suggesting cerebellar impairment can necessitate a switched handedness [21]). The tumor grew back when the patient was 8 years of age, leading to one subsequent procedure to remove the tumor (which also necessitated additional surgeries for shunt repairs and strabismus up until the patient was 12 years of age). At our assessment, the patient reported an unremitting impact of the tumor on her fine motor skills and on her ability to maintain good balance. Nonetheless, the patient reported a high level of academic success across her educational experiences. Despite noting that homework assignments took longer for her to complete than might be expected, she reported consistently receiving very superior scores on her coursework and that she had achieved high grade point averages in her secondary and graduate academic experiences.

Patient reported taking 20 mg of Celexa for the past four years for OCD and symptoms of depression. Patient stated that she had been experiencing symptoms of OCD since childhood, having been diagnosed with the condition by her neurologist when she was 8 years old. Of note, the patient reported that OCD runs in her father’s side of the family. Patient described her OCD as involving her brain “going on repeat,” “getting caught on things,” or “looping back” such that she was easily distracted by noises in her environment and had difficult focusing, leading her to continually check back and re-read every sentence that she would write. Patient reported experiencing “self-diagnosed anhedonia” since she was a college student though she noted that she had grown accustomed to these symptoms of low mood, stating that they did not interfere with her life. Patient also reported having non-diagnosed misophonia (e.g., strong reactions to particular sounds) since childhood. Patient had not recently received psychotherapy for these symptoms though she did see a pediatric psychologist beginning at age 5 and continuing until she was 9 years old. She also briefly received cognitive-behavioral therapy (CBT) for her OCD symptoms while in college though noted that she did not find the treatment to be particularly helpful. Patient stated that she performed ongoing “self-CBT” for her OCD, enabling her to function.

Patient reported having an “Asperger-like personality,” contributing to feelings of isolation that were consistently present in her life. Patient did not have many friends growing up and she reported not having any close social relationships. She attributed her lack of interest in socializing to the long time that it took her to complete tasks, leading her to feel a constant time pressure to accomplish the tasks that were most important to her. Nonetheless, patient considered herself to be socially adept despite not being interested in seeking social relationships. She reported learning several tricks to blend in and have normal surface-level interactions; these included asking others about themselves, having her body face another individual when they were communicating, and applying self-deprecating humor in conversation.

In her prior neuropsychological report, the patient was diagnosed with Axis I Learning Disorder, Not Otherwise Specified (NOS) (Nonverbal Learning Disability [NVLD]) and OCD. The NVLD diagnosis was largely based on the discrepancy between the patient’s verbal and spatial intellectual capacities as assessed by the Wechsler Adult Intelligence Scale-III (WAIS-III). OCD was not formally evaluated during the assessment process and was based on the patient’s prior diagnosis and on behavioral observations collected during the assessment process.

### Neuropsychological assessments

Table 1 depicts the outcomes for all neuropsychological assessments conducted during the patient’s four testing sessions at Boston University. Standard scores (also referred to as scaled scores) are included throughout Table 1 and represent conversions of the raw scores onto a common scale. The distribution of standard scores have a mean of 100 and a standard deviation of 15. We conducted assessments of intelligence and general achievement (e.g., Wechsler Adult Intelligence Scale-IV [WAIS-IV] [22], Woodcock Johnson IV Tests of Achievement [WJ-IV]) [23], social cognition (e.g., Advanced Clinical Solutions for the WAIS-IV [ACS]) [24], attention and executive functioning (e.g., Stroop test [25], Rey-Osterrieth Complex Figure Test [26, 27], Conners Continuous Performance Test 3 [CPT 3] [28], Delis-Kaplan Executive Function System Test [D-KEFS]) [29], memory (e.g., California Verbal Learning Test II [CVLT-II] [30], Wechsler Memory Scale-IV [WMS-IV]) [31], and motor functioning (e.g., Finger Tapping Test [32, 33], Grooved Pegboard Test [34]). For the purposes of this case report, we will focus on data from certain assessments.

**Table 1:** Neurocognitive test results

Neurocognitive test	Score	Population Mean (SD)	Clinical Significance^a^	
			≥ -1SD	≥ -2SD
WAIS-IV				
*Verbal Comprehension Index*	149	100 (15)		
Similarities	19	10 (3)		
Vocabulary	19	10 (3)		
Information	16	10 (3)		
*Perceptual Reasoning Index*	94	100 (15)		
Block Design	11	10 (3)		
Matrix Reasoning	8	10 (3)		
Visual Puzzles	8	10 (3)		
Figure Weights	16	10 (3)		
Picture Completion	7	10 (3)	√	
*Working Memory Index*	125	100 (15)		
Digit Span	14	10 (3)		
Arithmetic	15	10 (3)		
*Processing Speed Index*	105	100 (15)		
Symbol Search	12	10 (3)		
Coding	10	10 (3)		
Cancellation	2	10 (3)		√
Full Scale IQ	122	100 (15)		
WAIS-IV ACS				
Affect Naming	5	10 (3)		√
Faces I	8	10 (3)		
Faces II	13	10 (3)		
WJ-IV				
Passage Comprehension	106	100 (15)		
Calculation	117	100 (15)		
Sentence Reading Fluency	116	100 (15)		
Math Facts Fluency	116	100 (15)		
Sentence Writing Fluency	103	100 (15)		
Reading Recall	118	100 (15)		
Reading Vocabulary	130	100 (15)		
Stroop Test				
Word	25	50 (10)		√
Color	33	50 (10)	√	
Color-Word	60	50 (10)		
Interference	61	50 (10)		
Rey-Osterrieth Complex Figure Test				
Copy	≤ 1	Percentile		√
Immediate Recall	≤ 1	Percentile		√
Delayed Recall	≤ 1	Percentile		√
Organization	53	50 (10)		
Recognition Total Correct	54	50 (10)		
CPT-3				
Response Style	17	50 (10)		√
Detectability	58	50 (10)		
% Omissions	45	50 (10)		
% Commissions	76	50 (10)		√
Perseverations	48	50 (10)		
Hit Reaction Time	39	50 (10)	√	
Hit Reaction Time Standard Deviation	46	50 (10)		
Variability	47	50 (10)		
Hit Reaction Time Block Change	57	50 (10)		
Hit Reaction Time Interstimulus Interval Change	40	50 (10)		
D-KEFS Trail Making Test				
Visual Scanning	11	10 (3)		
Number Sequencing	7	10 (3)	√	
Letter Sequencing	8	10 (3)		
Number Letter Switching	11	10 (3)		
Motor Speed	1	10 (3)		√
CVLT-II				
Total Hits Trials 1-5	55	50 (10)		
Short Delay Free Recall	1.5	0 (1)		
Long Delay Free Recall	1	0 (1)		
Recognition	100%	Percent Recognized		
WMS-IV				
Symbol Span	13	10 (3)		
Spatial Addition	3	10 (3)		√
Visual Reproduction I	12	10 (3)		
Visual Reproduction II	8	10 (3)		
Visual Reproduction Recognition	3–9	Percentile	√	
Finger Tapping Test^b^				
Left hand (taps)	14.4	*M =* 49.0, *SD =* 4.1		√
Right hand (taps)	14.8	*M* = 44.6, *SD* = 4.6		√
Grooved Pegboard^b^				
Left hand (seconds)	105	*M* = 69.66, *SD* = 19.27	√	
Right hand (seconds)	180	*M* = 75.80, *SD =* 21.56		√

*WAIS-IV* Wechsler Adult Intelligence Scale-IV [22]; *WJ-IV* Woodcock Johnson IV Tests of Achievement [23], *ACS* Advanced Clinical Solutions for the WAIS-IV [24], *CPT 3* Conners Continuous Performance Test 3 [28], *D-KEFS* Delis-Kaplan Executive Function System Test [29], *CVLT-II* California Verbal Learning Test II [30], *WMS-IV* Wechsler Memory Scale-IV [31]

^a^Reflects clinically relevant results at two thresholds: greater than or equal to either one or two standard deviations more impaired than the mean. Scores that represent better performance than the mean are not indicated. CPT-3 Hit RT can be clinically significant in either direction from the mean (lower than mean is faster performance)

^b^Indicated means and SDs are from standardization samples

Patient’s performance on the WAIS-IV suggested a high level of general intellectual functioning with a Full-Scale Intelligence Quotient (FSIQ) in the 93th percentile, reflecting her overall superior cognitive capacities; of note, however, there was marked variability in her performance across domains of verbal comprehension, perceptual reasoning, working memory, and processing speed that comprise the FSIQ. Across the administered neuropsychological tests, patient’s performance in the domain of verbal reasoning and utilization of verbal content emerged as a personal strength, with the patient scoring in the 99.9th percentile on the Verbal Comprehension Index.

Patient’s performance on certain tests was consistent with impairments in visuospatial reasoning and fine motor skills, likely stemming from her cerebellar brain tumor. She exhibited her lowest WAIS-IV performance in the perceptual reasoning domain, scoring in the 34th percentile, though there was variability in the patient’s performance across the various subtests that comprise the domain. For instance, she exhibited average performance on the Block Design, Visual Puzzles, and Matrix Reasoning subtests (with her score on Block Design being superior to her performance on the former two subtests) and very superior performance on the Figure Weights subtest. Assessments supporting the patient’s visuospatial deficits included the WMS-IV Spatial Addition task (standard score of 3), WMS-IV Visual Reproduction tasks (particularly in correctly recognizing the originally presented figures, 3–9 percentile) and the Rey-Osterrieth Complex Figure Task (≤1 percentile in copy, immediate recall, and delayed recall). Several tests also confirmed the patient’s deficits in motor dexterity including the Finger Tapping Test and the Grooved Pegboard Test. On the Finger Tapping Test, across 5 trials on each hand, the patient exhibited approximately 14 taps over a 10-s duration; as a comparison, the age-matched normative sample mean across both hands ranges from approximately 45 taps to 49 taps. On the Grooved Pegboard Test, the patient exhibited several peg drops and, when using her left hand, completed the task in nearly 3 min; this was markedly slower than the normative, age-matched sample mean task duration of approximately 1 min. Of note, the patient’s low performance on the Rey-Osterrieth Complex Figure Task also may have served as an indication of fine motor deficits as the patient had some difficulty in drawing straight, non-interrupted lines while copying the original image. Overall, neuropsychological testing data supported the impact of the patient’s childhood cerebellar tumor on her present-day motor skills and visuospatial capabilities, warranting a diagnosis of major neurocognitive disorder due to another medical condition, with behavioral disturbance.

The patient’s perfectionistic, detail-oriented tendencies were apparent across several assessments. Most notable was the patient’s execution of the D-KEFS Trail Making Test Motor Speed Task which required her to trace a dotted line that connects several open circles as quickly as possible. During the practice trial, the patient asked the assessor whether she was supposed to connect each dash individually (e.g., from one dash to the next). The assessor informed the patient that she should instead focus on drawing the line as quickly as possible and not on connecting each individual dash. On the test round, the patient did not connect each dash individually but still completed the task in over 87 s (scaled score of 1), suggesting that her heightened focus on precision carried into the test administration. Other assessments revealed a pattern of over-responding such that the patient was “doing more” than she needed to for that particular task. For instance, on the CVLT-II, the patient demonstrated a high 16 repetitions during her recall across the 5 trials, reflecting her potential concern about missing a word. On the Conners CPT 3, the patient demonstrated a very elevated commission rate (e.g., responding to non-target letters), suggesting that she was worried about missing a letter as it flashed across the screen, thus leading her to over-respond to irrelevant stimuli. At other times, patient’s perfectionistic tendencies manifested as her focusing so closely on the details of tasks such that she lost sight of their intended meaning. On the WAIS-IV Picture Completion Task, when asked to identify the missing elements of the image, the patient’s responses suggested that she was focusing very closely on the fine details of the image and thus not able to adopt a broader perspective in determining the most essential missing component. For instance, when asked to identify what was missing in an image depicting a hand holding a rose, she stated that “the person is not grasping the rose tightly enough.” When she was asked what was missing in an image of a snowy landscape that featured a barn, the patient reported that barn doors typically have a second element on their door that allows the door to be opened or closed, as opposed to identifying the more essential missing piece.

We conducted a test of social cognition to provide further context for the patient’s self-reported “Asperger-like” personality and the patient’s prior NVLD diagnosis. The WAIS-IV ACS Affect Naming Test required the patient to correctly label the emotion being expressed by a target face from a list of seven possible emotions (happy, sad, angry, afraid, surprised, disgusted, and neutral). Out of 24 identification questions, the patient identified 9 incorrectly; this performance corresponded to the 5th percentile. Notably, 4 incorrect identifications involved the patient not recognizing when a face was “afraid” and 3 of these incorrect identifications involved the patient labeling a face as “angry” when the correct answer was “no feeling/neutral.” The ACS Faces Test involved cards featuring various faces, some of which were placed on a grid in front of the patient for several seconds. Once the face cards were removed, the patient was asked to select the previously presented cards and place them in the correct spots on the grid. Unlike the ACS Affect Naming Test, the ACS Faces Test did not involve emotion recognition. The patient performed in the average range on the ACS Faces Test immediate recall (Faces I; 25th percentile) and in the high average range on delayed recall (Faces II; 84th percentile).

### Clinical assessments and interview

We conducted two structured clinical assessments in order to gather additional clinical data relevant to the patient’s self-reported anhedonia and previously-diagnosed OCD. The Anxiety and Related Disorders Interview Schedule for DSM-5 (ADIS-5) [35] is a structured interview that evaluates current mood, anxiety, obsessive-compulsive, trauma, and related disorders. Upon formal assessment, patient did not meet criteria for OCD (endorsing neither compulsions nor obsessions) or major depressive disorder/persistent depressive disorder (with her only salient symptom in this domain being depressed mood). The Structured Clinical Interview for DSM-IV Axis II Personality Disorders (SCID-II) [36] is a structured assessment that provides a formal evaluation of personality disorders by inquiring on specific diagnostic criteria. Given the patient’s history of perfectionistic, obsessive-compulsive tendencies, we formally assessed for obsessive-compulsive personality disorder (OCPD). During this assessment, the patient reported that she was the kind of person to focus on order, organization, and details, noting that she liked to make schedules and lists. Patient reported having a mental checklist for her school work, her hygiene, and her food (e.g., ensuring that she followed a specific calorie count and ate certain nutrients). Patient reported sometimes getting so caught up in her focus on these areas that she lost sight of what she was trying to accomplish; she provided the example of putting on sunscreen in the morning and being very focused on not missing a spot such that she would realize after 20 min that she was still putting sunscreen on her face. She also reported having trouble finishing jobs “all the time” because she was focused on getting things exactly right and not missing anything; for instance, on an exam, she would spend a great deal of time on her responses to make sure that she did not make any mistakes, leading a test to take much longer than it would have if she had simply followed her instincts. Patient reported being so focused on certain domains of her life – specifically, her schoolwork, food consumption, hygiene, and regular exercise routine – such that she did not have much remaining time for pursuing friendships. Patient also reported experiencing some difficulty in discarding non-disposable items, largely attributed to the patient’s concern about having to buy something again at a later point, leading her apartment to be very cluttered as a result. Patient reflected that, as a child, she was very stubborn, had strong opinions, and talked back frequently. Overall, given the patient’s responses, we provided the patient with an OCPD diagnosis.

## Discussion and conclusions

This case study explores a patient presenting to our clinic with a childhood history of a cerebellar tumor that had contributed to an ongoing pattern of visuospatial impairments and fine motor skill deficits. Other patterns of the clinical presentation – specifically, the patient’s detail-oriented, perfectionistic tendencies, her self-described anhedonia, and her socio-emotional impairments – were less readily accounted for by her complex medical history. However, emerging data associating cerebellar damage with restricted socio-emotional processing and the onset of psychiatric conditions such as OCD, depressive disorders, and personality pathology may provide some additional insight on this case and on subsequent similar presentations. Indeed, this presentation extends the existing literature on this topic in many ways. First, whereas most prior case studies have generally focused on patients with current and/or recent acute cerebellar conditions in adulthood [7, 9], this case study centered on a patient with a childhood history of a cerebellar tumor and thus provides some insight on the ongoing impact of cerebellar damage even years after the initial medical event. Of note, one recent case study reported the onset of borderline personality disorder in a patient following the rupture of a cerebellum arteriovenous malformation that had been present since the patient’s childhood [15]. Though the lesion in this study occurred in adulthood, the presence of cerebellar abnormality in the patient’s youth is somewhat comparable to the present case study. Moreover, prior case studies have generally centered on individuals whose cerebellar impairment has resulted in overall cognitive slowing [7, 10]. Whereas the presented patient does have marked deficits in motor control and visuospatial processing, her superior cognitive and general intellectual functioning (e.g., WAIS-IV FSIQ in the 93rd percentile, VCI in the 99.9th percentile) is notable as is her self-reported high-level of academic achievement. Further, to our knowledge, our study is among the first to report on the potential association between cerebellar impairment and the onset of OCPD; of note, early signs of the patient’s detail-oriented, perfectionistic tendencies were present in her childhood, manifesting at the time as stubbornness (e.g., talking back to her mom) and thus providing further support for the possible role of the patient’s cerebellar tumor in her OCPD pathology. Notably, the link between cerebellar impairment and OCPD is in line with prior case studies which have noted increased rigidity of thought and stubbornness in patients with right-sided cerebellar lesions [7]. It should be noted that we did not have access to the patient’s medical records, thus precluding us from providing additional diagnostic information about the patient’s cerebellar tumor and associated procedures. The one other relevant case study that enrolled a patient with OCPD reported on an individual with a left cerebellar lesion who had a premorbid diagnosis of OCPD and late-onset OCD with the latter condition hypothesized to be linked to the cerebellar lesion given the patient’s age (e.g., as OCD after 50 generally stems from an organic basis [37]) [12]. Specifically, the authors suggested that the OCD may have developed from an interaction between the lack of “homeostatic control” created by the cerebellar lesion and the patient’s already-existent OCPD [12]. In contrast, the patient that we discuss in this case report was diagnosed with OCD when she was 8 years old, around the time of her second cerebellar tumor surgery, for a pattern of behaviors that included perseveration, excessive hand washing, and working on math problems for a long time even after she had already solved the problem. Such symptoms are reflective of the types of detail-oriented, perfectionistic tendencies that we noted in the patient throughout our assessment process, yet the patient did not meet current OCD diagnostic criteria. OCD is marked by the presence of obsessions (e.g., recurrent, persistent thoughts, images, or urges that the individual attempts to neutralize or suppress) or compulsions (e.g., repetitive behaviors or mental acts that the individual feels motivated to complete in response to an obsession) [38], neither of which the patient endorsed. OCPD is rooted in a preoccupation with perfectionism and orderliness, often while sacrificing efficiency and flexibility [38]; this pattern was reflective of the patient’s symptoms and thus informed our diagnostic choice of OCPD.

Further, despite the patient’s social skill deficits which are often seen in patients with NVLD [38], we felt that the patient’s visuo-spatial and fine motor deficits were better accounted for by an organic basis (e.g., her childhood brain tumor), thus leading us to instead diagnose her with a major neurocognitive disorder due to another medical condition. We added the “with behavioral disturbance” specifier to account for her impairments across other domains of functioning (e.g., social processing deficits). Our confidence in this diagnosis was enhanced by the fact that the patient’s previous NVLD diagnosis had been presumably entirely based on the discrepancy between her verbal and spatial abilities on the WAIS-III.

Our assessment process thus resulted in the changing of two diagnoses – 1) NVLD to major neurocognitive disorder due to another medical condition, with behavioral disturbance and 2) OCD to OCPD. The necessity of these diagnostic changes highlights the importance of exercising great caution when assigning diagnoses to patients with these types of complex neurological histories (e.g., cerebellar tumors) as symptoms can be overlapping across diagnostic conditions, making differentiation a challenge. For instance, it is not unusual for a patient to have comorbid OCD and OCPD with 23–32% of OCD patients also having a comorbid diagnosis of OCPD [12, 39]. Thus, it is possible that the symptoms of the one diagnosis could blend into the symptoms of the other diagnosis, potentially leading to misdiagnosis of a given patient’s condition. Proper diagnosis is essential so that a patient can be directed down an appropriate path of treatment, as opposed to a path that may not be necessary and/or advisable.

The relatively high comorbidity of OCD and OCPD suggest a possible link between these two conditions [12, 39, 40]. Given that the patient had a family history of OCD, it is possible that her past OCD and current OCPD presentations were compounded by her having learned modeled behavior and/or her having a genetic vulnerability. However, in light of the recent literature linking cerebellar impairment with emotion deficits and OCD pathology [12, 20], the patient’s illness presentation suggests the role of factors that extend beyond familial psychiatric history [7, 9, 10, 12].

At the conclusion of our assessment process, we provided the patient with specific recommendations for academic accommodations and subsequent clinical care. The patient’s marked deficits in visuospatial reasoning and fine motor skill domains warranted provision of continued academic accommodations including access to someone who could take notes for her, assistance with filling in the bubbles on multiple-choice tests, double time on assessments, restricted amount of writing and typing that she needed to complete, and presentation of visual information in a verbal format whenever possible. We felt that the patient’s perfectionistic, detail-oriented tendencies likely had an overall adverse impact on her task efficiency and cognitive flexibility, leading us to refer her for CBT at our clinic to help her better understand her perfectionistic inclinations and thus reduce their impact on her cognitive performance. We also noted that CBT would likely be useful for helping the patient with her ongoing, self-identified symptoms of anhedonia and in supplying her with social skills training that could enhance the quality of her relationships with others.

Our case study provides a glimpse into the clinical presentation of a high-functioning individual with a childhood brain tumor associated with characteristic present-day deficits (e.g., impaired visuospatial processing and poor fine motor control) and a unique array of additional clinical characteristics (e.g., detail-oriented, perfectionistic tendencies, self-reported anhedonia, blunted socio-emotional processing). Despite the patient’s superior level of overall daily functioning, her marked adult impairments in emotional and social processing suggest that a childhood cerebellar tumor may leave an imprint across multiple domains. Thus, our findings suggest the importance of early, intensive psychotherapy, social skills training, and cognitive rehabilitation as possible preventive measures in the immediate aftermath of cerebellar damage.

## Abbreviations

ACS: Advanced Clinical Solutions for the WAIS-IV
ADIS-5: Anxiety and Related Disorders Interview Schedule for DSM-5
CPT 3: Conners Continuous Performance Test 3
CVLT-II: California Verbal Learning Test II
D-KEFS: Delis-Kaplan Executive Function System Test
SCID-II: Structured Clinical Interview for DSM-IV Axis II Personality Disorders
WAIS-IV: Wechsler Adult Intelligence Scale-IV
WJ-IV: Woodcock Johnson IV Tests of Achievement
WMS-IV: Wechsler Memory Scale-IV
